# Delayed Hypersensitivity Reaction to Insulin: A Case Report

**DOI:** 10.7759/cureus.108303

**Published:** 2026-05-05

**Authors:** Mohammed Aharmim, Mohamed Lakhal, Nezha Reguig, Jamal Eddine El Bourkadi

**Affiliations:** 1 Pulmonology and Phthisiology, Moulay Youssef Hospital, Rabat, MAR; 2 Pulmonology, Centre Hospitalier Universitaire (CHU) Ibn Sina, Rabat, MAR; 3 Respiratory Diseases, Centre Hospitalier Universitaire (CHU) Mohammed VI, Faculty of Medicine and Pharmacy, Mohammed I University, Oujda, MAR; 4 Pulmonology, Centre Hospitalier Universitaire (CHU) Ibn Sina, Rabat, MAR, Rabat, MAR

**Keywords:** delayed hypersensitivity, desensitization, insulin allergy, metacresol, type 1 diabetes mellitus, type 1 diabetes mellitus (t1d)

## Abstract

Advances in the purification of animal insulin preparations and the introduction of recombinant human insulin have markedly reduced the frequency of insulin hypersensitivity reactions. However, preservatives such as metacresol and other excipients contained in insulin formulations may still induce hypersensitivity reactions, which may be immediate, corresponding to type I hypersensitivity, or delayed, suggesting type III or, more commonly, type IV hypersensitivity according to the Gell and Coombs classification. We report the case of a 25-year-old woman with a 2-year history of type 1 diabetes mellitus who was initially treated with insulin detemir and insulin glulisine. Fifteen months after the initiation of insulin therapy, she developed delayed erythematous skin lesions associated with mild headaches and abdominal pain. Several alternative insulin preparations were subsequently tried, but all reproduced the same symptoms despite antihistamine therapy. Dose splitting and rotation of injection sites failed to improve the reaction. Specific IgE antibodies to human insulin, porcine insulin, protamine, and latex were negative. Skin prick tests performed with different insulin preparations were negative on immediate reading; however, 12 hours later, the patient developed a diffuse and extensive cutaneous reaction, including facial involvement. Because all tested insulin preparations triggered a reaction, their excipient profiles were reviewed, and metacresol was identified as the only common excipient among them. As no metacresol-free insulin preparation was available in Morocco, tolerance induction with insulin was undertaken in an attempt to induce tolerance despite probable hypersensitivity to metacresol. The procedure was successful and resulted in sustained tolerance to all insulin preparations. Insulin allergy remains a rare yet challenging condition that requires careful diagnostic evaluation. Identification of the causative mechanism, including possible hypersensitivity to excipients such as metacresol, is crucial to guide appropriate management.

## Introduction

Advances in the purification of animal insulin preparations and the development of recombinant human insulin have substantially reduced the prevalence of insulin hypersensitivity reactions, now estimated at 0.1% to 3% [1-4]. However, preservatives such as metacresol and excipients used to prolong insulin action, including protamine and zinc, may also induce hypersensitivity reactions [1,2]. These reactions may be immediate and IgE-mediated (type I hypersensitivity) or delayed, reflecting type III or type IV hypersensitivity according to the Gell and Coombs classification [1,2]. Clinical manifestations range from localized skin reactions to life-threatening anaphylaxis [1]. Management can be challenging and often requires a multidisciplinary approach [1,3].

We report a case of delayed hypersensitivity reaction to insulin with the probable involvement of metacresol, successfully managed with tolerance induction [1,4].

## Case presentation

We report the case of a 25-year-old woman with a 2-year history of type 1 diabetes mellitus. She had no personal history of drug allergy, atopy, or autoimmune disease. Her family history was notable only for a grandmother with type 2 diabetes mellitus.

She was initially treated with insulin detemir and insulin glulisine. Fifteen months after the initiation of insulin therapy, she developed delayed cutaneous reactions consisting of erythematous lesions occurring 10 to 12 hours after insulin injection, associated with mild headache and abdominal pain. There was no vomiting, diarrhea, or loss of consciousness.

**Figure 1:**
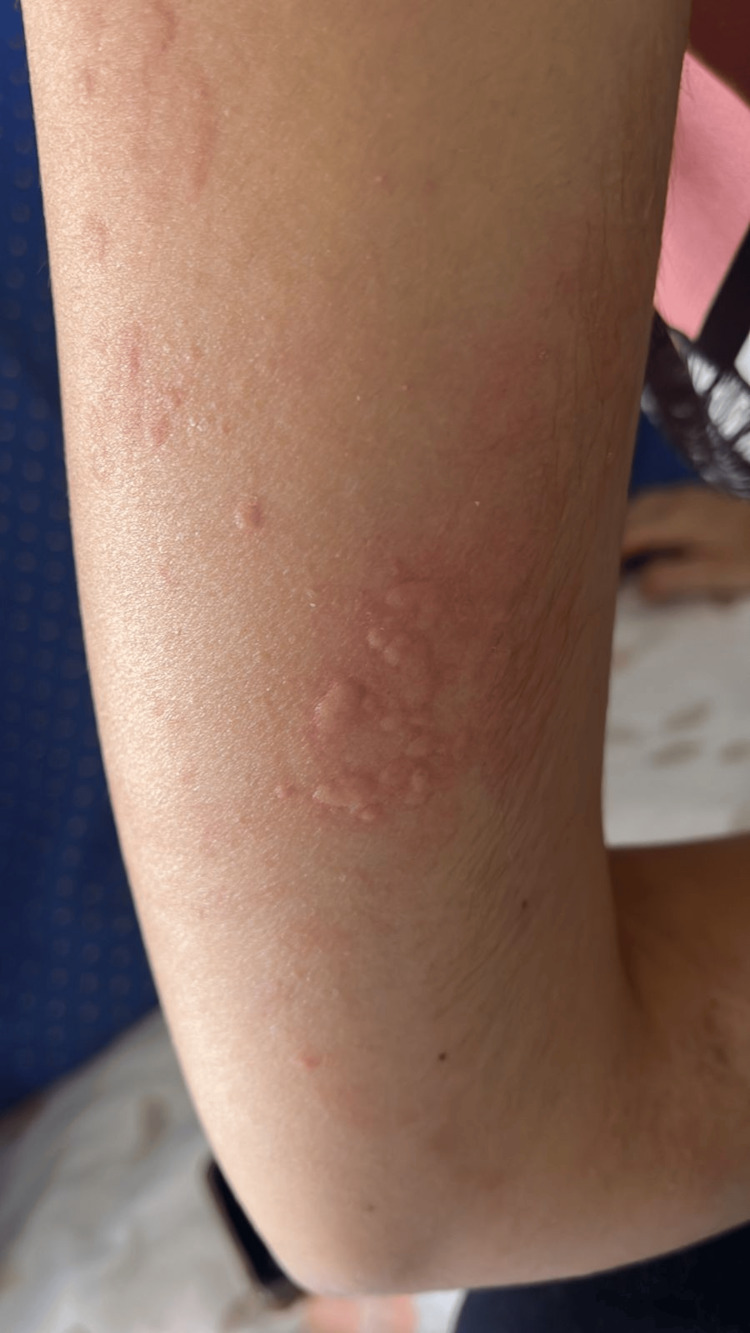
Erythematous papular skin reaction involving the upper limb

The lesions persisted for one to two weeks before resolving completely. They were initially confined to the injection sites and later extended to the upper and lower limbs (Figure 1). No co-factors such as physical exercise or concomitant medications, particularly nonsteroidal anti-inflammatory drugs, were identified at the time of these reactions.

Several alternative insulin preparations were subsequently prescribed by her endocrinologist, including insulin glargine, insulin lispro, biphasic insulin aspart, insulin glargine U-300, NPH insulin, biphasic human insulin, and insulin aspart. However, all of them reproduced the same symptoms despite antihistamine therapy.

On examination, the patient was in good general condition, with a body mass index of 21 kg/m². Her diabetes was poorly controlled, with a glycated hemoglobin level of 11.2%, because she had become nonadherent to insulin therapy due to fear of recurrent hypersensitivity reactions. Complete blood count showed no eosinophilia.

Dose splitting and rotation of injection sites did not improve the reaction. Local contributing factors, including poor injection technique, inappropriate use of local disinfectants, and contact allergy to disinfectants, were considered unlikely. Specific IgE antibodies to human insulin, porcine insulin, protamine, and latex were all negative.

Skin prick testing was then performed using 0.05 mL of different undiluted insulin preparations, including insulin degludec, NPH (neutral protamine Hagedorn) insulin, insulin glargine U-300, insulin glargine, insulin degludec/insulin aspart, insulin aspart, insulin lispro, regular human insulin, biphasic insulin aspart, and biphasic human insulin. Normal saline and histamine were used as negative and positive controls, respectively. Metacresol, protamine sulfate, and zinc chloride were not tested separately because these agents were not available in our country.

**Figure 2:**
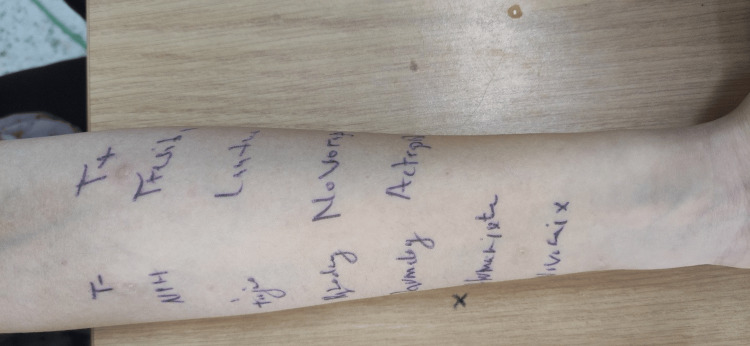
Skin tests with different insulin preparations were negative on immediate reading.

**Figure 3:**
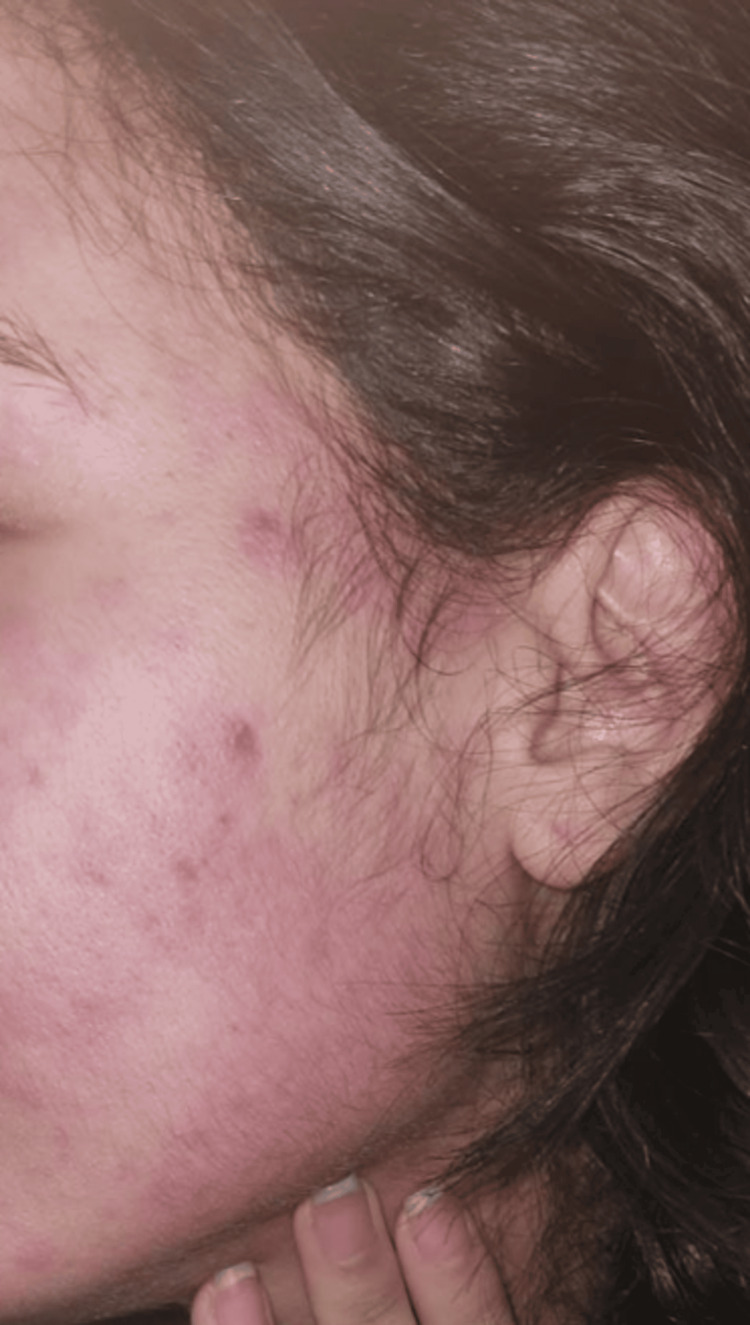
Cutaneous reaction involving the face

Immediate readings of the skin prick tests were negative (Figure 2). However, 12 hours later, the patient developed a diffuse and extensive cutaneous reaction, including facial involvement. Delayed formal reading of the tests could not be performed because the patient had to travel unexpectedly for family reasons. Fortunately, she was able to document the reaction with photographs (Figure 3).

Given this generalized reaction induced by skin prick testing, we decided not to perform delayed intradermal testing with the different insulin preparations. Patch testing was also not performed because it was not available locally. Because the patient reacted to all insulin preparations, we reviewed the excipient profiles of each product and found that metacresol was the only common excipient identified among the tested preparations. Since metacresol was shared by all formulations and no alternative explanation was identified, it was considered the most likely cause of the hypersensitivity reaction.

As therapeutic options were limited and no metacresol-free insulin preparation was available in Morocco, tolerance induction with insulin was undertaken in an attempt to induce tolerance despite probable hypersensitivity to metacresol, although the delayed chronology of the reaction suggested a type IV hypersensitivity mechanism. A slow desensitization protocol over seven days was selected under coverage with methylprednisolone, montelukast, and cetirizine. An initial dilution of 1 unit of insulin in 100 mL of 0.9% sodium chloride was administered, with gradual dose escalation every 6 hours over a total period of 7 days. Intravenous administration was used during the first two days, followed by subcutaneous administration starting on day 3. Details of the desensitization protocol are summarized in Table 1.

**Table 1:** Insulin desensitization protocol Human insulin doses used in the seven-day desensitization protocol

Time	Day 1	Day 2	Day 3	Day 4	Day 5	Day 6	Day 7
8:00	0.00001	0.0001	0.001	0.01	0.1	1	8
14:00	0.00002	0.0002	0.002	0.02	0.2	2	8
20:00	0.00004	0.0004	0.004	0.04	0.4	4	8

The procedure successfully induced tolerance to all insulin preparations. The patient was maintained on montelukast and cetirizine for one month, and after one year of follow-up, all insulin preparations remained well-tolerated.

## Discussion

Although recombinant human insulin has significantly reduced hypersensitivity reactions, such reactions still occur and may target either insulin or excipients such as metacresol, zinc, and protamine [1,2]. While immediate IgE-mediated reactions are more commonly reported, delayed hypersensitivity reactions, including type III and type IV mechanisms, are increasingly recognized [1,2].

Delayed type IV reactions are often associated with non-insulin excipients, particularly metacresol [2,5-8]. These reactions typically present as localized indurated lesions appearing several hours after injection and may occasionally be associated with systemic symptoms [5,8].

Cases of leukocytoclastic vasculitis induced by insulin have also been described [6]. Additionally, metacresol allergy has been associated with both local and systemic manifestations, including gastrointestinal and autonomic symptoms [5,7].

The diagnostic workup of delayed insulin hypersensitivity relies on delayed-reading skin tests and patch testing, although evidence regarding their sensitivity and safety remains limited [1,9]. In our case, delayed reactions following skin testing limited further evaluation.

Management depends on the underlying mechanism. Immediate reactions may be treated with switching insulin preparations, desensitization, or biologic therapies such as omalizumab and rituximab [1,3,10]. In contrast, delayed hypersensitivity reactions are more difficult to manage, with limited standardized approaches [4].

In our setting, the absence of metacresol-free insulin represented a major limitation. Therefore, a desensitization strategy was adopted. Intravenous insulin administration has been reported to induce immunologic tolerance and may be less likely to trigger hypersensitivity reactions [10].

The successful outcome in our patient supports the use of tolerance induction in selected cases. The clinical presentation strongly suggests a probable metacresol-induced delayed hypersensitivity reaction.

## Conclusions

Insulin allergy remains a rare but challenging condition that requires careful diagnostic evaluation. Identification of the causative mechanism, including possible hypersensitivity to excipients such as metacresol, is essential to guide appropriate management. Management relies on exclusion of differential diagnoses, therapeutic adaptation, and, when necessary, desensitization as an effective and potentially safe option.
